# Revision of the Afrotropical genus *Ivondrovia* Shenefelt & Marsh, 1976 with description of a new species (Hymenoptera: Braconidae: Doryctinae)

**DOI:** 10.3897/zookeys.747.24351

**Published:** 2018-04-02

**Authors:** Sergey Belokobylskij, Alejandro Zaldivar-Riveron, Ruben Castañeda-Osorio

**Affiliations:** 1 Museum and Institute of Zoology of the Polish Academy of Sciences, Wilcza 64, 00–679 Warsaw, Poland; 2 Zoological Institute of the Russian Academy of Sciences, Universitetskaya naberezhnaya 1, Saint Petersburg 199034, Russia; 3 Colección Nacional de Insectos, Instituto de Biología, Universidad Nacional Autónoma de México, 3er. circuito exterior s/n, Cd. Universitaria, Copilco, Coyoacán, A. P. 70–233, C. P. 04510., D. F., México

**Keywords:** Descriptions, Doryctinae, Holcobraconini, key to species, *Lophogaster*, parasitoid

## Abstract

A revision of the small Afrotropical holcobraconine genus *Ivondrovia* Shenefelt & Marsh, 1976 (Doryctinae) is provided. A new species from Kenya, *Ivondrovia grangeri* **sp. n.**, is described and illustrated. The illustrated redescriptions of the genus *Ivondrovia* and its type species *Lophogaster seyrigi* Granger, 1949 are given. The two known species of this genus are keyed.

## Introduction

The doryctine genus *Ivondrovia* Shenefelt & Marsh, 1976 was originally described from Madagascar by Granger (1949) as *Lophogaster* Granger, 1949 (a primary homonym for *Lophogaster* Sars, 1857 of Crustacea) only containing its type species, *L. seyrigi* Granger, 1949. In the World Catalogue of Braconidae, Shenefelt and Marsh (1976) subsequently changed this preoccupied name to *Ivondrovia*. This taxon was originally included by Granger (1949) in the subfamily Odontobraconinae which is now regarded as the tribe Holcobraconini within the braconid subfamily Doryctinae (Belokobylskij 1992; Belokobylskij et al. 2004; Zaldívar-Riverón et al. 2008).

For the reclassification of subfamily Doryctinae, Belokobylskij (1992) examined the holotype of *Ivondrovia* type species, *L. seyrigi*, and classified *Ivondrovia* within the monotypic subtribe Ivondroviina belonging to the tribe Holcobraconini. The Ivondroviina was characterized by the following combination of characters: hind coxa simple (without dorsal projections); occipital carina and frons keel absent; propodeum with distinct medio-posterior areola and basal carinae but without total foveolate sculpture; first flagellar segment of antenna not longer than second one; recurrent vein (1m-cu) of fore wing postfurcal in comparison with the first radiomedial vein (2RS); radial vein (RS) of hind wing arising from basal vein (1r-m) and rather far from costal vein (SC+R); second metasomal tergite long medially and laterally and very short sublaterally.

The biology of *Ivondrovia* is still unknown. However, since the majority of the Doryctinae are known as ectoparasitoids of bark boring and xylophagous beetle larvae, and several holcobraconine species belonging to the genera *Zombrus* Marshall, 1897 and *Odontobracon* Cameron, 1887 were reared from large Capricorn beetle larvae, it is likely that species of *Ivondrovia* also infest larvae of the beetle family Cerambycidae for their development.

During ongoing work on the molecular phylogeny of the tribe Holcobraconini, the authors discovered a second new species of *Ivondrovia* from Kenya. Here we describe this new species and provide a key for determination of the two known species of *Ivondrovia*.

## Materials and methods

The terminology employed for morphological features and measurements follows Belokobylskij and Maetô (2009). The wing venation nomenclature follows Belokobylskij and Maetô (2009), with Sharkey and Wharton (1993) terminology shown in parentheses. Specimens were examined using a MC–2 stereomicroscope. Photographs were taken with a Leica IC 3D digital camera mounted on a Leica MZ16 microscope and using the Leica Application Suite imaging system at the Museum and Institute of Zoology, Warsaw, Poland. The photograph images were enhanced and plate was composed using Adobe Photoshop.

The specimens examined in this study are deposited in the following collections: Colección Nacional de Insectos, Instituto de Biología, Universidad Nacional Autónoma de México (**UNAM**), Muséum National d’Histoire Naturelle, Paris, France (**MNHN**), and Zoological Institute of the Russian Academy of Sciences, St Petersburg, Russia (**ZISP**).

## Taxonomy

### Genus Ivondrovia

Taxon classificationAnimaliaHymenopteraBraconidae

Shenefelt & Marsh, 1976

Lophogaster Granger, 1949: 93.
Ivondrovia Shenefelt & Marsh 1976: 1364; Belokobylskij 1992: 912; Yu et al. 2012.

#### Type species.

*Lophogaster seyrigi* Granger, 1949.

#### Description.

*Head* (Figs 3–6, 22–25), not depressed, high, transverse. Ocelli arranged in slightly obtuse triangle. Frons slightly concave, with low double longitudinal carinae in anterior third (between ocellar sockets), slightly convex medially. Eyes glabrous. Occipital carina completely absent; occiput medio-dorsally with distinct, short, and divergent postero-laterally after short base hook (Fig. 4). Malar suture absent. Clypeus slightly convex, with distinct and rather long lower flange, almost entirely delineated from face by shallow or very shallow furrow. Hypoclypeal depression medium size and subrounded. Postgenal bridge very narrow. Maxillary palpus rather long (Fig. 2), 6-segmented, its sixth (apical) segment almost as long as fifth segment, third segments slightly widened and without projection in lower margin; labial palpus (Fig. 2) short, 4-segmented, its second segment slightly thickened, third segment not shortened, 0.8 times as long as fourth segment. Antennae weakly setiform. Scape of antenna (Figs 7, 26) wide and rather short, without apical lobe or basal constriction, with deep emargination in inner upper margin, its ventral margin (lateral view) not longer than dorsal margin. First flagellar segment sub-cylindrical, on outer side straight, almost as long as second segment.

*Mesosoma* (Figs 8, 9, 28, 29) not depressed. Neck of prothorax short. Pronotum dorsally weakly convex, its anterior flange short and curved upwards; pronope absent, pronotal carina distinct and slender, placed submedially on pronotum (dorsal view). Propleural dorsoposterior flange long and partly medially wide. Mesoscutum highly and convex-roundly elevated above pronotum. Median lobe of mesoscutum without median longitudinal furrow and anterolateral corners. Notauli deep and complete. Tegula (Fig. 27) distinctly widened distally, convex along outer margin. Prescutellar depression (scutellar sulcus) long and deep, with one high median carina. Scuto-scutellar suture distinct. Scutellum almost flat, with weak lateral carinae. Metanotum without median longitudinal carina. Sternaulus (precoxal sulcus) very shallow, narrow, long, straight, smooth. Prepectal carina distinct and complete, rather wide ventrally, prolonged laterally till upper margin of sternaulus. Postpectal carina absent. Metapleural flange long, wide, subtriangular, rounded apically. Propodeum with areas distinctly delineated by carinae, mainly smooth, with high sublateral carinae; lateral tubercles indistinct, propodeal bridge absent. Propodeal spiracles rather big and bean-shape. Metapleural suture very shallow.

*Wings* (Figs 12, 14–16, 31–33). Pterostigma of fore wing rather narrow. Radial vein (r) arising distinctly before middle of pterostigma. Radial (marginal) cell distinctly shortened. Both radiomedial veins (2RS and r-m) present. Second radiomedial (submarginal) cell medium length. First (r) and second (3RSa) radial abscissae forming an obtuse angle. Recurrent vein (1m-cu) slightly postfurcal or almost interstitial. Nervulus (1cu-a) postfurcal. Discoidal (first discal) cell petiolate anteriorly, petiole (1RS) not long and slightly thickened. Parallel vein (2CUb) arising from posterior 0.2 of apical margin of brachial (second subdiscal) cell. Brachial (second subdiscal) cell closed postero-apically by short, sclerotised and slightly inclivous brachial vein (2cu-a). Transverse anal veins (1a and 2a) absent. Hind wing with three hamuli (Fig. 32). First abscissa of costal vein (C+Sc+R) long, 0.8 times as long as second abscissa (SC+R); first abscissa (C+Sc+R) not divided apically on two branches. Radial vein (RS) arising from basal vein (1r-m) rather far from costal vein (SC+R). Radial (marginal) cell distinctly narrowed posteriorly, closed before apex of wing, without additional transverse vein (r). Medial (basal) cell not widened towards apex, subparallel-sided in apical half, 12.5 times longer than wide, 0.5 times as long as hind wing. Nervellus (cu-a) present, slightly sinuate. Submedial (subbasal) cell long. First abscissa of mediocubital vein (M+CU) 0.9–1.0 times as long as second abscissa (M) (till basal vein (1r-m)). Recurrent vein (m-cu) strongly antefurcal, very long, strongly curved in posterior 0.7 towards apex of wing, divergent apically with medial vein (2M).

*Legs*. Fore femur thick and short. Fore tibia with numerous short and thick spines arranged in wide vertical stripe (Figs 11, 30). Fore tarsus 1.5–1.6 times longer than fore tibia. Segments of middle tarsus short. Middle tibiae thick, with almost single line of thick spines. Hind coxa (Fig. 13) rather wide and short, without basoventral tooth and corner, dorsally without any processes or teeth, but distinctly convex. Fore and middle femora with low dorsal protuberances; hind femur without dorsal protuberance, thick. Hind tibia thick. Hind tibial spurs long, almost straight. Basitarsus of hind tarsus approx. half as long as second-fifth segments combined. Claws large, short, strongly curved, simple.

*Metasoma* (Figs 17–19, 34, 37, 38). First tergite sessile, short, and wide, strongly widened subbasally. Acrosternite of first segment short, 0.15 times as long as first tergite. Dorsope of first tergite small; basolateral lobes present, narrow and directed below; spiracular tubercles absent, spiracles situated in basal 0.3 of tergite; with distinct thick and slightly convergent posteriorly sub-lateral carinae and wide median carinae formed from two carinae curvedly fused in basal fifth of tergite. Second tergite with deep, wide, short, slightly divergent posteriorly and fused with second suture lateral furrows; these furrows and second suture medially define a large and leaf-shaped median area; additionally, second tergite in basal half with sub-triangular area and slightly separated by shallow or considerably shallow furrows. Second suture deep, rather wide, very strongly curved medially, sublaterally with very deep and sharp bends. Third tergite with shallow submedian transverse depression. Second-sixth tergites with separate laterotergites. Fourth-sixth tergites in their basal halves covered by rather sparse and long pale setae. Hypopygium medially on posterior margin with short and subpointed process. Ovipositor apically with two obtuse and weak dorsal nodes (Figs 20, 36). Ovipositor sheath longer than metasoma (Fig. 21).

#### Hosts.

Unknown.

#### Distribution.

Afrotropical Region.

##### Key to *Ivondrovia* species

**Table:** 

1	Hind tibia entirely and most part of hind tarsus light reddish-brown. Head mainly yellow. Fore wing faintly infuscate, with yellow tint especially basally. Fore tibia with two lines of strong spines. Nervellus (cu-a) of hind wing subperpendicular to mediocubital vein (M+CU). Second tergite weakly punctate-rugulose on leaf-shaped median area. Third and fourth tergites of female sub-posteriorly without narrow transverse punctate furrows. Body length 7.2–9.7 mm. Madagascar	***I. seyrigi* (Granger, 1949)**
–	Hind tibia and hind tarsus entirely black. Head mainly light reddish-brown. Fore wing entirely strongly infuscate, without yellow tint. Fore tibia with tree lines of strong spines. Nervellus (cu-a) of hind wing distinctly oblique to mediocubital vein (M+CU). Second tergite strongly striate on leaf-shaped median area. Third and fourth tergites of female sub-posteriorly with narrow transverse punctate furrows. Body length 7.3–7.9 mm. Kenya	***I. grangeri* sp. n.**

### Ivondrovia grangeri sp. n.

Taxon classificationAnimaliaHymenopteraBraconidae

http://zoobank.org/11847BE7-9D54-4145-9242-14B868FCF6F6

Figs 1–11 , 12–20

#### Type material.

**Holotype**: female, “Kenya: Nyanza Prov., Gembe Hills in dry gallery woodland Olea europaea ssp. cuspidata common, 1362 m, 0°29.36’S, 34°15.60’E, 22–29.i.2005, R. Copeland”, “CNIN 3090” (ZISP). **Paratypes**. 1 female, with same first label, “CNIN 3326” (UNAM); 1 female, with same first label, but “8–15.i.2005”, “CNIN 3609” (UNAM); 1 female, Kenya, “Nyanza Prov. Gembe Hills in dry gallery woodland Olea europaea ssp. cuspidata common, 1362 m, 0°29.36’S – 34°15.60’E, R. Copeland” (MNHN).

#### Comparative diagnosis.

This new species is very similar to *I. seyrigi*; the differences between both species are indicated in the foregoing key.

#### Description.

Female. Body length 7.3–7.9 mm; fore wing length 5.7–5.8 mm.

*Head* width 1.60–1.75 times its median length, 1.15–1.20 times width of mesoscutum. Occiput distinctly concave. Head behind eyes (dorsal view) slightly convex anteriorly and weakly roundly narrowed posteriorly. Transverse diameter of eye 1.2–1.3 times longer than temple. Ocelli enlarged, in triangle with base 1.1–1.2 times its sides, situated on median line of eyes. POL 1.10–1.25 times Od, 0.30–0.35 times OOL. Eye without emargination opposite antennal sockets, 1.1–1.2 times as high as broad. Malar space 0.4 times as high as eye, 0.75–0.80 times as high as basal width of mandible. Face width 1.3–1.4 times height of eye and 1.50–1.65 times height of face and clypeus combined. Clypeus almost flat (lateral view). Width of hypoclypeal depression 0.8–0.9 times distance from edge of depression to eye, 0.4–0.5 times width of face. Hypostomal flange narrow.

*Antenna* thickened, almost filiform, 51-segmented. Scape 1.5–1.7 times longer than its maximum width. First flagellar segment 2.4–2.6 times longer than its apical width, 0.90–0.95 times as long as second segment. Submedian segments 1.5 times longer than their widths.

*Mesosoma*. Length 1.7 times its height. Lateral side of pronotum without longitudinal carina, mainly smooth. Median lobe of mesoscutum distinctly convex, protruding forwards and weakly rounded anteriorly. Notauli entirely deep and distinct, rather wide, smooth. Prescutellar depression (scutellar sulcus) deep, long, only with strong median carina, smooth, 0.3–0.4 times as long as scutellum. Scutellum 1.15–1.20 times longer than its maximum width. Subalar depression shallow, entirely smooth. Precoxal sulcus (sternaulus) shallow, narrow, smooth, connected anteriorly with prepectal carina, running along all lower part of mesopleuron. Metapleural lobe without dense pubescence along posterior margin. Propodeum (lateral view) distinctly and evenly convex.

*Wings*. Fore wing 3.4–3.5 times longer than its maximum width. Pterostigma 3.5–4.2 times longer than width. Metacarpus (R1a) 1.1–1.2 times longer than pterostigma, 1.5–2.0 times longer than distance between apex of radial (marginal) cell and apex of wing. Radial vein (r) arising from basal 0.4 of pterostigma. Second radial abscissa (3RSa) 1.9–2.1 times longer than first abscissa (r), 0.4 times as long as the slightly, evenly curved third abscissa (3RSb), 1.3–1.5 times longer than the almost straight and oblique first radiomedial vein (2RS). Second radiomedial (submarginal) cell slightly widened towards apex, 2.6–2.9 times longer than its maximum width, 0.85 times as long as the rather wide brachial (first subdiscal) cell. Brachial (first subdiscal) cell slightly convex anteriorly. First medial abscissa ((RS+M)a) slightly sinuate. Recurrent vein (1m-cu) 1.1–1.2 times longer than first radiomedial vein (2RS), 0.6 times as long as basal vein (1M); recurrent (1m-cu) and basal (1M) veins subparallel. Discoidal (first discal) cell long, 2.5–2.6 times longer than its maximum width. Distance from nervulus (1cu-a) to basal vein (1M) 0.25–0.30 times nervulus (1cu-a) length. Hind wing 3.6 times longer than its maximum width. First abscissa of mediocubital vein (M+CU) 0.9–1.0 times as long as second abscissa (1M); basal part of second abscissa (1M) (before recurrent vein (m-cu)) 3.5 times longer than apical part of second abscissa (1M) (behind recurrent vein (m-cu)). Recurrent vein sclerotised, blackish.

*Legs*. Hind coxa 1.2–1.4 times longer than maximum width, 0.80–0.85 times as long as propodeum. Hind femur 2.4–2.6 times longer than width. Hind tarsus 0.9–1.0 times as long as hind tibia. Second segment of hind tarsus 0.5 times as long as basitarsus, 0.75–0.80 times as long as fifth segment (without pretarsus).

*Metasoma* 1.2 times longer than head and mesosoma combined. First tergite rather strongly and obliquely widened basally, then distinctly and weakly-roundly widened from almost base to subapex, slightly narrowed apically, without oblique apico-lateral furrows. Maximum width of first tergite 1.5–1.6 times its width at dorsope level, 2.1–2.3 times its minimum width; length 1.1 times its apical width. Median length of second tergite 0.5 times its basal width, 2.3–2.5 times length of third tergite. Ovipositor sheath almost equal to metasoma, 1.7 times longer than mesosoma, 0.7 times as long as fore wing.

*Sculpture and pubescence*. Head (including frons and clypeus) smooth. Mesosoma mainly smooth; mesoscutum in medioposterior third with longitudinally median carina and striation on rather wide area, metapleuron posteriorly punctate. Propodeum with medial length and subtriangular or suboval areola situated in posterior 0.4–0.5 of segment, with coarse long mediobasal carina in anterior half of segment, basolateral areas almost fused posteriorly with apico-lateral areas, mainly smooth. Hind coxa and femur entirely smooth. First tergite almost smooth widely in basal two-thirds and mostly laterally, distinctly striate in medio-apical third, with distinct longitudinal medial carina narrowly branched in apical half, lateral carinae slightly convergent. Second tergite medially strongly striate on large leaf-shaped median area, densely and distinctly punctate laterally; third tergite sparsely punctate and partly smooth, with crenulated and rather narrow transverse submedian furrow. Third and fourth tergites sub-posteriorly with transverse narrow punctate furrows. Remaining tergites distinctly sparsely punctate, smooth posteriorly. Vertex almost entirely glabrous, usually with not long and semi-erect setae laterally. Mesoscutum mainly glabrous, with rather dense, short and semi-erect pale setae laterally and in medioposterior third. Mesopleuron widely glabrous medially. Hind tibia dorsally with rather dense, short, and semi-erect golden setae, ventrally with dense or very dense, short and semi-erect golden setae and additionally with sparse, long and semi-erect setae; length of setae on dorsal margin of tibia 0.3–0.5 times maximum width of hind tibia.

*Colour*. Body almost entirely light reddish brown, head with large black spot on all or most part of frons and on median part of vertex. Antenna entirely black. Palpi reddish brown, darker basally. Legs light reddish brown or partly reddish brown, apical segments of fore and middle tarsi dark brown to black; hind tibia and tarsus entirely black, tarsus sometimes medially dark reddish brown. Ovipositor sheaths black. Fore and hind wings entirely distinctly infuscate, without yellowish tint. Pterostigma entirely black.

**Figures 1–11.**
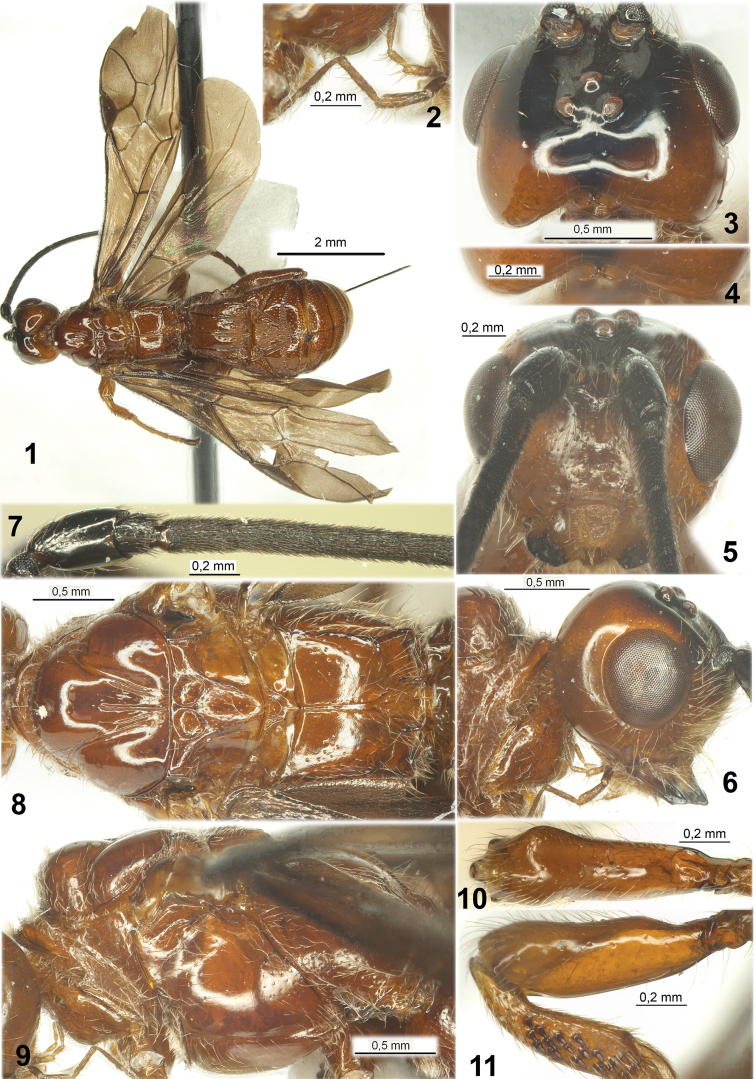
*Ivondrovia grangeri* sp. n., female, holotype. **1** habitus, dorsal view **2** palpi **3** head, dorsal view **4** upper median process of occiput **5** head, front view **6** head, lateral view **7** basal segments of antenna **8** mesosoma, dorsal view **9** mesosoma, lateral view **10** fore femur, dorsal view **11** fore femur and tibia, lateral view.

**Figures 12–20.**
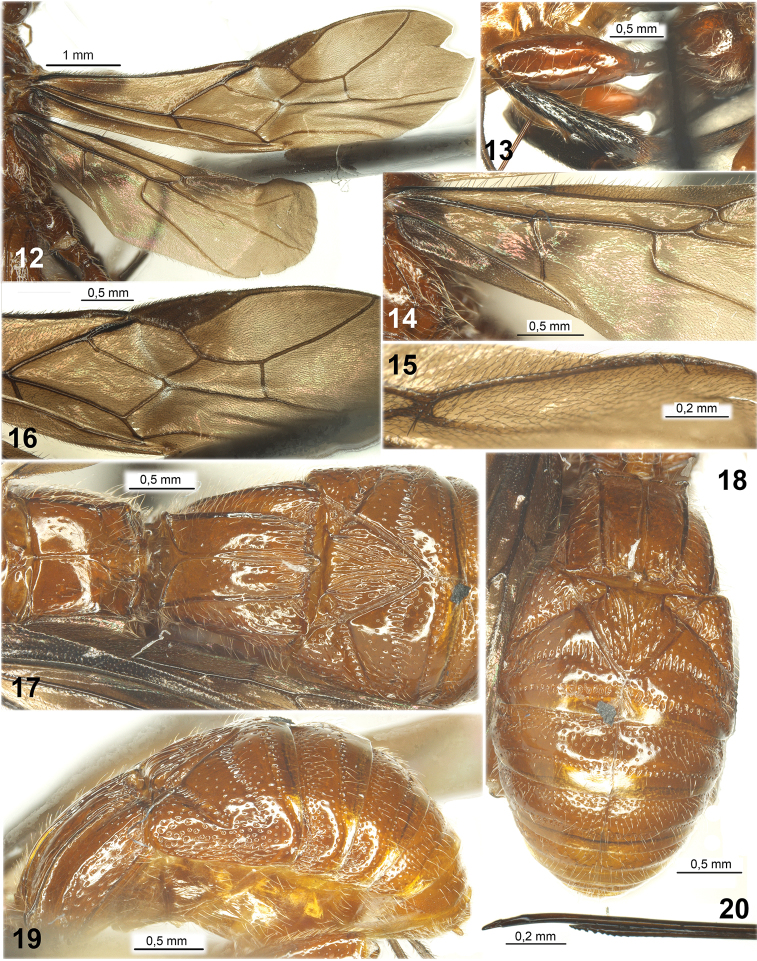
*Ivondrovia grangeri* sp. n., female, holotype. **12** fore and hind wings **13** hind leg **14** basal part of hind wing **15** upper apical part of hind wing **16** apical part of fore wing **17** propodeum and three basal tergites of metasoma, dorsal view **18** metasoma, dorsal view **19** metasoma, lateral view **20** apex of ovipositor, lateral view.

#### Male.

Unknown.

### Ivondrovia seyrigi

Taxon classificationAnimaliaHymenopteraBraconidae

(Granger, 1949)

Figs 21–30 , 31–38

Lophogaster seyrigi Granger, 1949: 93.
Ivondrovia seyrigi (Granger, 1949): Shenefelt and Marsh 1976: 1364; Yu et al. 2005.

#### Material examined.

Type specimens. Lectotype: female, “Madagascar, Ivondro”, “Museum Paris, I. 39”, “Type”, “♀ *Lophogaster seyrigi* Granger, C. van Achterberg, 1980, Lectotype” (MNHN). Paralectotype: 1 male, “Madagascar, Bekily, reg. sud de l’ile”, “Museum Paris, III.37, A. Seyrig”, “♂ *Lophogaster seyrigi* Granger, C. van Achterberg, 1980, Paralectotype” (MNHN).

Non-type specimen: 1 male, “Madagascar, Bekily, reg. sud de l’ile”, “Museum Paris, I.39, A. Seyrig” (MNHN).

#### Comparative diagnosis.

The differences between the type species and *I. grangeri* sp. n. are given in the above-mentioned key.

#### Description.

Female. Body length 9.7 mm; fore wing length 8.0 mm.

*Head* width 1.5 times its median length, 1.1 times width of mesoscutum. Occiput strongly concave. Head behind eyes (dorsal view) slightly convex anteriorly and slightly roundly narrowed posteriorly. Transverse diameter of eye 1.45 times longer than temple. Ocelli enlarged, in triangle with base 1.1 times its sides, situated distinctly before median line of eyes. POL 0.8 times Od, 0.25 times OOL. Eye without emargination opposite antennal sockets, 1.15 times higher than broad. Malar space 0.45 times as high as eye, 0.8 times as high as basal width of mandible. Face width 1.4 times height of eye and 1.5 times height of face and clypeus combined. Clypeus almost flat (lateral view). Width of hypoclypeal depression equal to distance from edge of depression to eye, 0.4 times width of face. Hypostomal flange narrow.

*Antenna* thickened, weakly setiform, more than 35-segmented (apical segments missing). Scape 1.6 times longer than its maximum width. First flagellar segment slightly thickened, 2.0 times longer than its apical width, almost as long as second segment. Submedian segments 1.8 times longer than their width.

*Mesosoma*. Length 1.8 times its height. Lateral side of pronotum without longitudinal carina, mainly smooth. Median lobe of mesoscutum distinctly convex, protruding forwards and weakly rounded anteriorly. Notauli entirely deep, but slightly less deep posteriorly, narrow, almost smooth. Prescutellar depression (scutellar sulcus) deep, long, only with median carina, smooth, 0.3 times as long as scutellum. Scutellum 1.1 times longer than its maximum width. Subalar depression entirely smooth. Precoxal sulcus (sternaulus) very shallow, connected anteriorly with prepectal carina, running along all lower part of mesopleuron. Metapleural lobe without dense pubescence along posterior margin. Propodeum (lateral view) rather distinctly broken submedially.

*Wings*. Fore wing 3.2 times longer than its maximum width. Pterostigma 4.5 times longer than its width. Metacarpus (R1a) 0.9 times as long as pterostigma, 1.7 times longer than distance between apex of radial (marginal) cell and apex of wing. Radial vein (r) arising from basal 0.35 of pterostigma. Second radial abscissa (3RSa) 1.8 times longer than first abscissa (r), 0.4 times as long as the distinctly curved third abscissa (3RSb), 1.5 times longer than the almost straight and oblique first radiomedial vein (2RS). Second radiomedial (submarginal) cell slightly widened towards apex, 2.4 times longer than its maximum width, 0.75 times as long as the rather narrow brachial (second discal) cell. Brachial (second discal) cell slightly convex anteriorly. First medial abscissa ((RS+M)a) slightly sinuate. Recurrent vein (1m-cu) 1.25 times longer than first radiomedial vein (2RS), 0.65 times as long as basal vein (1M); recurrent (1m-cu) and basal (1M) veins subparallel. Discoidal (first discal) cell long, 2.7 times longer than its maximum width. Distance from nervulus (1cu-a) to basal vein (1M) 0.25 times nervulus (1cu-a) length. Hind wing 4.2 times longer than its maximum width. First abscissa of mediocubital vein (M+CU) 0.9 times as long as second abscissa (1M); basal part of second abscissa (1M) (before recurrent vein (m-cu)) approx. twice longer than apical part of second abscissa (1M) (behind recurrent vein (m-cu)). Recurrent vein (m-cu) sclerotised, yellowish.

*Legs*. Middle tarsus 1.2 times longer than middle tibia. Hind coxa 1.2 times longer than maximum width, 0.85 times as long as propodeum. Hind femur 2.5 times longer than its width. Hind tarsus 0.9 times as long as hind tibia. Second segment of hind tarsus 0.5 times as long as basitarsus, 0.8 times as long as fifth segment (without pretarsus).

*Metasoma* almost as long as head and mesosoma combined. First tergite strongly and obliquely widened basally, then distinctly and weakly-roundly widened from subbase to subapex, slightly narrowed apically, without oblique furrows apico-laterally. Maximum width of first tergite 1.4 times its width at dorsope level, 1.9 times its minimum width; length almost equal to its apical width. Median length of second tergite 0.6 times its basal width, 2.3 times length of third tergite. Ovipositor sheath 1.15 times longer than metasoma, 1.5 times longer than mesosoma, 0.7 times as long as fore wing.

*Sculpture and pubescence*. Head (including frons and clypeus) smooth. Mesosoma mainly smooth (mesoscutum medioposteriorly broken by pin), metapleuron posteriorly punctate-striate. Propodeum with short and semi-round areola situated in posterior third of segment, with coarse long mediobasal carina in anterior two-thirds of segment, basolateral areas long, fused with apico-lateral areas, mainly smooth. Hind coxa and femur entirely smooth. First tergite weakly punctate-rugulose and slightly smooth in basal half and laterally, distinctly striate in medio-apical half. Second and third tergites rather distinctly and sparsely punctate, smooth posteriorly. Remaining tergites mainly smooth. Vertex with rather sparse, long and almost erect setae marginally, widely glabrous medially. Mesoscutum mainly glabrous, with rather sparse, short and semi-erect pale setae along notauli and laterally. Mesopleuron widely glabrous medially. Hind tibia dorsally with dense short and semi-erect yellow setae, ventrally with very dense, short, and semi-erect golden setae and additionally with sparse, long and semi-erect setae; length of setae on dorsal margin 0.3–0.4 times maximum width of hind tibia.

*Colour*. Head yellow, with black wide spot on most part of frons and on median part of vertex. Mesosoma brownish yellow, mesoscutum darker. Metasoma light reddish brown, first tergite yellow (basally) to brownish yellow or light reddish brown (apically). Antenna entirely black. Palpi reddish brown, darker basally. Legs brownish yellow to light reddish brown, apex of hind tibia, all hind tarsus and apical segments of fore and middle tarsi brown or reddish brown. Ovipositor sheaths dark brown to black. Fore wing entirely distinctly infuscate with yellowish tint (especially basally). Pterostigma entirely brown.

**Figures 21–30.**
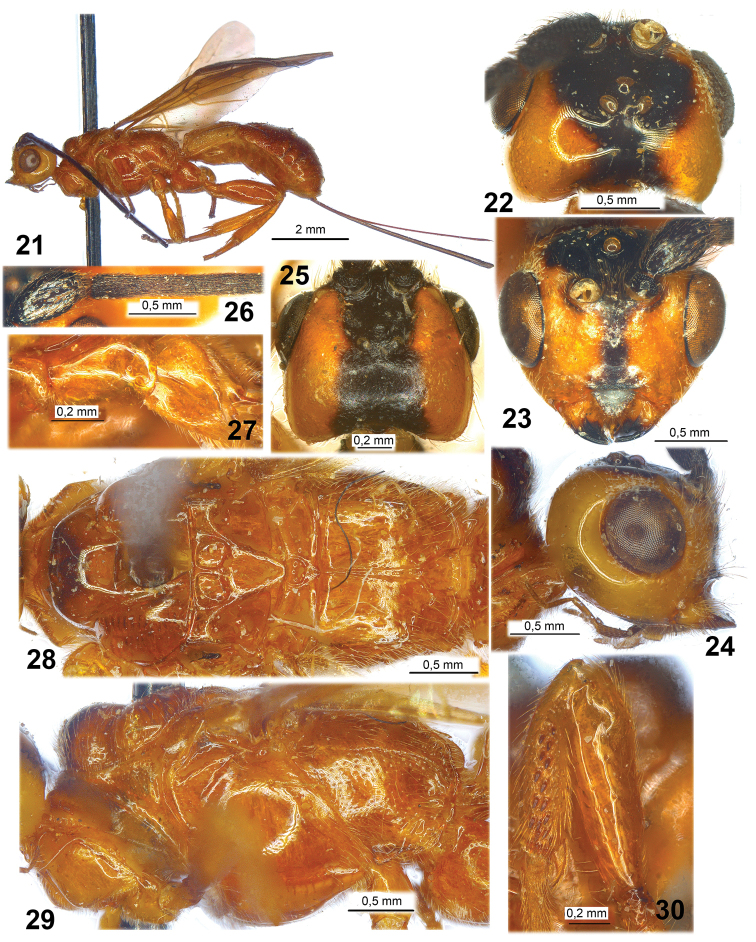
*Ivondrovia seyrigi* (Granger, 1949) (**21–24, 26–30** female, holotype; **25**, male, specimen). **21** habitus, lateral view **22**, **25** head, dorsal view **23** head, front view **24** head, lateral view **26** basal segments of antenna **27** tegula **28** mesosoma, dorsal view **29** mesosoma, lateral view **30** fore femur and tibia, lateral view.

**Figures 31–38.**
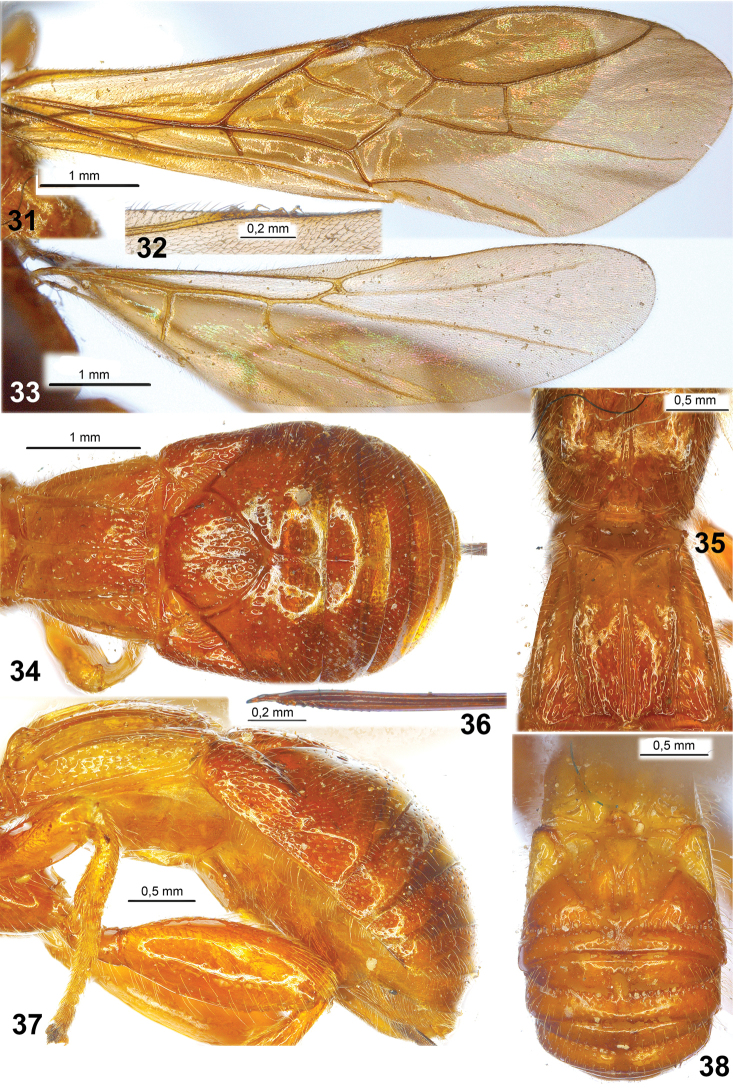
*Ivondrovia seyrigi* (Granger, 1949) (**31–37**, female, holotype; **38**, male, specimen). **31** fore wing **32** hamuli of hind wing **33** hind wing **34**, **38** metasoma, dorsal view **35** propodeum and first tergites of metasoma, dorsal view **36** apex of ovipositor, lateral view **37** metasoma, lateral view.

#### Male.

Body length 7.2 mm; fore wing length 5.5 mm. Head width 1.25 times its median length. Temple long; transverse diameter of eye 1.1 times longer than temple. Malar space 0.3 times as high as eye, 0.5 times as high as basal width of mandible. Face medially weakly and densely granulate, dark brown to black. Antenna 57-segmented, 1.6 times longer than body. Penultimate segment 2.3 times longer than wide, 0.7 times as long as apical segment; apical segment distinctly pointed. Anterior part of mesosoma brown to dark brown. First metasomal tergite elongate, 1.4 times longer than apical width; its sublateral carinae parallel. Second tergite with median triangular area distinctly separated by deep grooves, prolonged apically by distinct obtuse carina. Transverse groove on third tergite deep, wide and coarsely crenulated. Fourth to sixth tergites with deep, compete, crenulated and slightly curved transverse submedian furrow. Metasoma mainly smooth. Otherwise similar to female.

## Supplementary Material

XML Treatment for Ivondrovia

XML Treatment for Ivondrovia grangeri

XML Treatment for Ivondrovia seyrigi
